# Identifying potential causal effects of age at menarche: a Mendelian randomization phenome-wide association study

**DOI:** 10.1186/s12916-020-01515-y

**Published:** 2020-03-23

**Authors:** Maria C. Magnus, Anna L. Guyatt, Rebecca B. Lawn, Annah B. Wyss, Katerina Trajanoska, Leanne K. Küpers, Fernando Rivadeneira, Martin D. Tobin, Stephanie J. London, Debbie A. Lawlor, Louise A. C. Millard, Abigail Fraser

**Affiliations:** 1 MRC Integrative Epidemiology Unit at the University of Bristol, Bristol, UK; 2 Population Health Sciences, Bristol Medical School, Bristol, UK; 3 Centre for Fertility and Health, Norwegian Institute of Public Health, P.O. Box 222 Skøyen, 0213 Oslo, Norway; 4 Department of Health Sciences, University of Leicester, Leicester, UK; 5 School of Experimental Psychology, University of Bristol, Bristol, UK; 6 Epidemiology Branch, National Institute of Environmental Health Sciences, National Institutes of Health, Department of Health and Human Services, Research Triangle Park, NC USA; 7 Department of Internal Medicine, Erasmus MC, University Medical Center, Rotterdam, The Netherlands; 8 Division of Human Nutrition and Health, Wageningen University & Research, Wageningen, The Netherlands; 9 National Institute for Health Research, Leicester Respiratory Biomedical Research Centre, Glenfield Hospital, Leicester, UK; 10 NIHR Bristol Biomedical Research Centre at the University Hospitals Bristol NHS Foundation Trust and the University of Bristol, Bristol, UK; 11 Intelligent Systems Laboratory, Department of Computer Science, University of Bristol, Bristol, UK

**Keywords:** Menarche, Mendelian randomization, MR-pheWAS

## Abstract

**Background:**

Age at menarche has been associated with various health outcomes. We aimed to identify potential causal effects of age at menarche on health-related traits in a hypothesis-free manner.

**Methods:**

We conducted a Mendelian randomization phenome-wide association study (MR-pheWAS) of age at menarche with 17,893 health-related traits in UK Biobank (*n* = 181,318) using PHESANT. The exposure of interest was the genetic risk score for age at menarche. We conducted a second MR-pheWAS after excluding SNPs associated with BMI from the genetic risk score, to examine whether results might be due to the genetic overlap between age at menarche and BMI. We followed up a subset of health-related traits to investigate MR assumptions and seek replication in independent study populations.

**Results:**

Of the 17,893 tests performed in our MR-pheWAS, we identified 619 associations with the genetic risk score for age at menarche at a 5% false discovery rate threshold, of which 295 were below a Bonferroni-corrected *P* value threshold. These included potential effects of younger age at menarche on lower lung function, higher heel bone-mineral density, greater burden of psychosocial/mental health problems, younger age at first birth, higher risk of childhood sexual abuse, poorer cardiometabolic health, and lower physical activity. After exclusion of variants associated with BMI, the genetic risk score for age at menarche was related to 37 traits at a 5% false discovery rate, of which 29 were below a Bonferroni-corrected *P* value threshold. We attempted to replicate findings for bone-mineral density, lung function, neuroticism, and childhood sexual abuse using 5 independent cohorts/consortia. While estimates for lung function, higher bone-mineral density, neuroticism, and childhood sexual abuse in replication cohorts were consistent with UK Biobank estimates, confidence intervals were wide and often included the null.

**Conclusions:**

The genetic risk score for age at menarche was related to a broad range of health-related traits. Follow-up analyses indicated imprecise evidence of an effect of younger age at menarche on greater bone-mineral density, lower lung function, higher neuroticism score, and greater risk of childhood sexual abuse in the smaller replication samples available; hence, these findings need further exploration when larger independent samples become available.

## Background

Menarche (onset of menses) is a hallmark event in a woman’s life. Using conventional multivariable regression, a younger age at menarche has been associated with higher risk of death from all causes [1], death from cardiovascular disease [1, 2], reproductive cancers [3, 4], and depression [5], but lower risk of osteoporosis/fractures [6, 7]. It has been proposed that the well-known inverse association between childhood body mass index (BMI) and age at menarche explains some of the observed associations [8, 9]. However, due to the strong tracking of BMI across the life-course, it is challenging to disentangle the role of BMI as a potential confounder, as opposed to a mediator of associations between age at menarche and health outcomes [9]. It therefore remains unclear whether the previously reported associations between age at menarche and health outcomes reflect a causal effect.

One way of evaluating causality is to use single nucleotide polymorphisms (SNPs) as instrumental variables for the exposure of interest (here age at menarche), under the assumption that the allocation of SNPs at conception is random and unrelated to potential confounding factors [10]. Mendelian randomization has previously been used to explore effects of age at menarche on cardiometabolic traits [9, 11, 12], depression [13, 14], breast cancer [15, 16], educational level [17], lung function [18], osteoporosis [19], fracture risk [20], and reproductive/behavioral outcomes [21]. However, these have focused on hypothesized effects by exploring whether associations that have been widely examined in the literature have causal evidence from Mendelian randomization analyses. This approach can miss novel potentially important (unknown and unthought of effects). A hypothesis-free approach specifically aims to go beyond that narrow focus to gain new knowledge. For example, hypothesis-free genome-wide association studies, in comparison to candidate/hypothesized genetic association studies, have efficiently identified novel biological understanding. It is possible that a similar approach to a large group of non-genetic outcomes could yield novel causal understanding. Previous phenome-wide Mendelian randomization studies of BMI and smoking have provided novel evidence of effects on outcomes not previously identified as being associated with these exposures [22, 23]. Furthermore, as with previous candidate gene-association studies, previous Mendelian randomization studies of hypothesized associations had small sample sizes. The large sample size used here supports more precise estimates than these previous studies as well as the potential for novel etiological understanding.

The objective of this study was to systematically investigate causal effects of age at menarche on health-related traits, by conducting a Mendelian randomization phenome-wide association study (MR-pheWAS).

## Methods

### UK Biobank

**Fig. 1:**
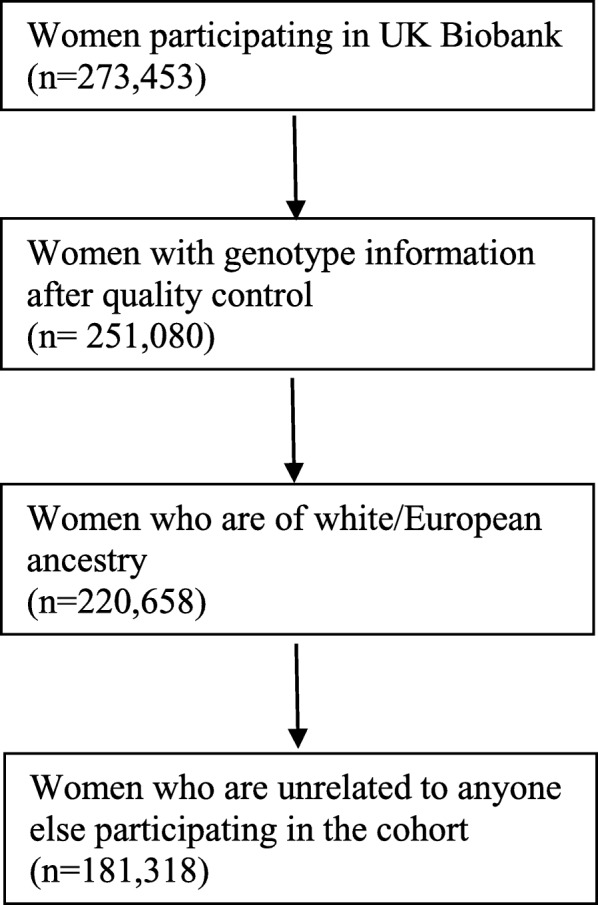
Illustration of the study population

The MR-pheWAS was undertaken in the UK Biobank cohort. The UK Biobank cohort includes 503,325 people (273,453 women) between 40 and 69 years of age, who were recruited between 2006 and 2010, from 22 assessment centers across England, Scotland, and Wales [24, 25]. The response rate was 5%, and all participants gave written informed consent. Participants were followed prospectively after enrolment using Hospital Episode Statistics data, as well as data from cancer registries and the Office of National Statistics. Age at menarche in whole years was self-reported at the time of enrolment (mean 44 years after the event). Genotyping was performed using the Affymetrix UK BiLEVE Axiom array on an initial 50,000 participants; the remaining 450,000 participants were genotyped using the Affymetrix UK Biobank Axiom® array [26]. Quality control and imputation (to over 90 million SNPs, indels, and large structural variants) was performed by the Wellcome Trust Centre for Human Genetics [26, 27]. The data collection in UK Biobank was approved by the NHS National Research Ethics Service (ref 11/NW/0382). The current analysis included 181,318 unrelated genotyped women of European ancestry (Fig. 1). Relatedness was defined as third degree relatives or closer [27].

### Identifying genetic instruments for age at menarche

We used the findings from the most recent genome-wide association study (GWAS) of age at menarche to identify genetic instruments for our analysis [16]. This study included 40 studies from the ReproGen consortium (*N* = 179,117 women), in addition to 23andMe (*N* = 76,831 women) and UK Biobank (*N* = 73,397 women). They identified 37,925 variants associated with age at menarche (*P* value < 5 × 10^−8^) that replicated across independent datasets, which constituted 389 independent signals. We generated an externally weighted genetic risk score (GRS_-all_) as a weighted sum of the number of age at menarche decreasing alleles across 360 SNPs, weighted by the published GWAS effect estimates. The weights that we used included a subpopulation of women participating in UK Biobank included in our MR-PheWAS. Specifically, up to 73,397 (40%) of the 181,318 women included in our analyses contributed to the age at menarche GWAS effect estimates. Thus, a higher GRS_-all_ reflects a younger age at menarche.

**Fig. 2:**
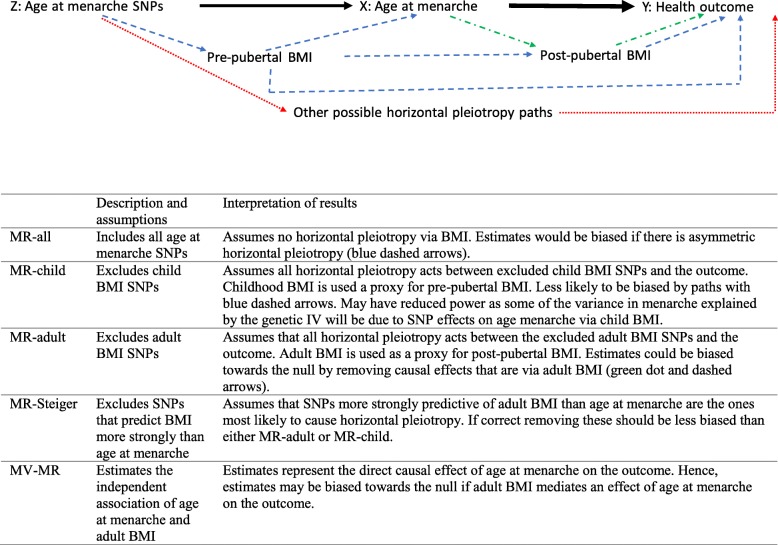
Directed acyclic graph. MR, Mendelian randomization; BMI, body mass index; MV, multivariable. We use hypothesis-free MR to explore the potential effect of age at menarche (X) on outcomes (Y), by using SNPs that robustly relate to age at menarche as instrumental variables (Z). The directed acyclic graph shows our key assumptions for the different genetic risk scores we use in our analyses. The black lines show this main analysis; the heavily weighted black line indicated the effects we are interested in. The MR assumption that Z does not relate to Y other than through X may be violated because of the known associations between some of the age at menarche SNPs and BMI. The genetic instrument Z could be associated with the outcome due to horizontal pleiotropy via child BMI, either via its relation to adult BMI or directly (blue dashed arrows). These paths could bias our MR results. Z could also be related to BMI via vertical pleiotropy through a path from Z to X, from X to adult BMI, and from it to Y (green dashed-dotted arrows). This path implies adult BMI is part of the causal path from age at menarche to Y and would not bias our results. We used four approaches to exploring these possibilities (table). We have not systematically explored other horizontally pleiotropic pathways that could bias our results (red dotted line)

We hypothesized a priori that there could be horizontal pleiotropic effects via childhood BMI, and that there could plausibly be horizontal or vertical pleiotropic effects via adult BMI, which is itself influenced by childhood BMI (Fig. 2). Horizontal pleiotropy could bias our findings. By contrast, vertical pleiotropy is part of the potential causal path between age at menarche and health outcomes that we are aiming to estimate. We used Steiger filtering to identify SNPs that explained more of the variation in adult BMI than the variation in age at menarche [28]. These SNPs are more likely to bias findings due to horizontal pleiotropy via BMI than SNPs that explain more of the variation of age at menarche. We generated a GRS (GRS_Steiger_) excluding the SNPs (27 SNPs) that explained more of the variation (quantified by the R^2^) in adult BMI than age at menarche. We only had a measure of adult BMI available in UK Biobank. Furthermore, 2 recent GWAS studies identified 15 and 941 SNPs associated with childhood and adult BMI, respectively [29, 30]. Of the 360 age at menarche SNPs included in our GRS_-all_, 7 and 199 SNPs were identified in, or situated near to (defined as being within 500,000 bp), SNPs identified in these childhood and adult BMI GWAS, respectively. We therefore also generated 2 alternative age at menarche GRS, excluding the 7 childhood BMI (GRS_-child BMI_) and all 206 BMI (GRS_-BMI_) associated SNPs, respectively.

### Phenome-wide Mendelian randomization analysis

We conducted a MR-pheWAS using the publicly available PHEnome Scan ANalysis Tool (PHESANT) version 0.17 which uses an automated rule-based method [31]. The decision rules start with identifying continuous, ordered categorical, unordered categorical, or binary variable fields. After outcome pre-processing (continuous traits were inverse normal rank transformed to ensure they were normally distributed), PHESANT runs linear (continuous outcomes), logistic (binary outcomes), ordered logistic (ordered categorical outcomes), and multinomial logistic (unordered categorical outcomes) regression, with the weighted allele score for age at menarche as the exposure. For unordered categorical outcomes analyzed using multinomial logistic regression, no beta coefficient or standard error is reported, but only the overall *P* value for the association based on a likelihood ratio test. All analyses are adjusted for age and the first 10 genetic principal components. The latter are used to account for population structure, which could produce confounded estimates.

We used PHESANT to examine the association of the age at menarche GRS with 17,893 health-related traits. To identify potential causal effects of age at menarche, we used 2 approaches that account for the number of tests performed, to help us evaluate the strength of the evidence from our MR-pheWAS. First, we derived a *P* value threshold setting the false discovery rate at 5%. After ranking the results by *P* value, this threshold is calculated as *P*_*t*_ (rank) = 0.05 × rank/*n*, where *n* is the total number of tests in the phenome scan and rank is the largest rank position with a *P* value less than *P*_*t*_. Second, we calculated a Bonferroni-corrected *P* value threshold, by dividing 0.05 by the number of tests performed. The Bonferroni-corrected *P* values assume that the tests conducted are independent. It is therefore likely to be an overly conservative correction for multiple testing, as several of the health-related traits evaluated are known to be associated. We reran our MR-pheWAS using our three alternative GRS_-Steiger_, GRS_-child BMI_, and GRS_-BMI_ scores as instruments. We used quantile-quantile (QQ) plots to illustrate how the distribution of the *P* values followed the distribution expected under the null hypothesis.

### Follow-up and replication analyses

We conducted follow-up and replication analyses of bone-mineral density (BMD), lung function, neuroticism, and childhood sexual abuse. The estimated relationships between the GRS_-all_ and these outcomes were all below the Bonferroni-corrected *P* value threshold in our main MR-pheWAS including all SNPs in the genetic risk score. They were further selected because of the novelty (not previously studied in detail using Mendelian randomization) and pragmatically—due to the availability of data to explore replication and potential assumption violations. Furthermore, we specifically chose not to follow-up any of the observed relationships with cardiometabolic health outcomes due to the close relationship with BMI.

We used information from 5 different cohorts/consortia to replicate our findings. These included 2 published GWAS studies of femoral neck (*n* = 22,990), lumbar spine (*n* = 22,177), and heel (*n* = 4566) BMD from the Genetic Factors for Osteoporosis (GEFOS) consortium [32, 33]; 2 GWAS studies of lung function (1 from the Cohorts for Heart and Aging Research in Genetic Epidemiology (CHARGE) consortium (*n* = 60,552) [34] and 1 from the SpiroMeta consortium (*n* = 79,055) [35]); and 1 GWAS study of neuroticism from the Genetics of Personality (GPC) consortium (*n* = 63,661) [36]. Replication analysis of childhood sexual abuse was done in the Avon Longitudinal Study of Parents and Children (ALSPAC, *n* = 5953) [37]. Detailed information of the replication cohorts and the definition of the outcomes used in the one- and two-sample Mendelian randomization analysis in both UK Biobank and the replications cohorts is provided in Additional file 1.

The first step in our follow-up of the findings in the MR-pheWAS was to estimate the magnitude of the causal effects. While estimates from PHESANT measure the association of the GRS with the outcomes (estimates reflect the mean difference or log-odds in outcomes per unit increase in the GRS), one- and two-sample Mendelian randomization analyses are required to estimate the magnitude of the causal effect of the exposure (per year decrease in age at menarche). We estimated the magnitude of potential causal effects of age at menarche on BMD, lung function, and neuroticism in both UK Biobank and the replication cohorts using one- and two-sample Mendelian randomization. We estimated the effect of each age at menarche SNP on these outcomes, then generated Wald ratios (the effect estimate of each SNP on the outcome divided by the effect estimate of each SNP on age at menarche), and subsequently pooled these SNP-specific estimates using random-effects inverse-variance weighting (equivalent to random-effects meta-analysis). We used one-sample Mendelian randomization analysis to estimate the magnitude of the causal effect of age at menarche on childhood sexual abuse in both UK Biobank and ALSPAC. This approach entailed first estimating the genetically predicted age at menarche from a linear regression model including the weighted GRS as a predictor (independent variable). We then used this genetically predicted age at menarche as the exposure in a logistic regression model of childhood sexual abuse. We estimated the standard errors of the second step using bootstrapping. The potential causal effects were estimated using the GRS_-all_, GRS_-Steiger_, and GRS_-BMI_ as instruments.

Secondly, we conducted a number of sensitivity analyses in the two-sample Mendelian randomization analysis to evaluate the assumption of no unbalanced horizontal pleiotropic effects that underlies the approach (Fig. 2). We estimated the effect using Mendelian randomization-Egger (MR-Egger) regression. The estimate of the causal effect from the MR-Egger regression is unbiased if the strength of the gene-exposure association does not correlate with the strength of the bias due to horizontal pleiotropy (known as the Instrument Strength Independent of Direct Effect, or InSIDE assumption) [38]. A non-zero intercept from this regression model is an indicator of unbalanced horizontal pleiotropy [38]. Additional sensitivity analyses included simple and weighted mode-based and weighted median regression [39]. We also used the Mendelian randomization pleiotropy residual sum and outlier (MR-PRESSO) test to identify possible bias from horizontal pleiotropy [40]. This includes a global test which evaluates the overall evidence of horizontal pleiotropy, an outlier-corrected causal estimate which corrects for the detected horizontal pleiotropy, and a distortion test which estimates if the causal effect estimate is different before and after adjustment for outliers (with a *P* value of < 0.05). We present the outlier-adjusted causal estimates for relationships where both the global and distortion tests provide evidence of horizontal pleiotropy (with both *P* values < 0.05).

To further examine the role of adult BMI on the observed relationships with age at menarche, we also conducted multivariable Mendelian randomization in UK Biobank, which is conceptually equivalent to using traditional multivariable regression techniques to identify the independent associations between multiple exposures and an outcome of interest (see Additional file 1) to estimate effects of age at menarche independent of adult BMI (i.e., the effect of age at menarche on the health-related traits not via BMI). We also adjusted for height using multivariable Mendelian randomization (the genetic risk score for height included 3285 variants identified in the most recent GWAS) in the analyses of lung function [30].

While one-sample and two-sample Mendelian randomization have the same three key assumptions, two-sample Mendelian randomization approaches vary in the extend and ways that the second assumption can be violated, which means that comparing results from both approaches is useful. In one-sample Mendelian randomization, causal estimates are robust to misspecification of the SNP-exposure association model (i.e., presence of interactions or nonlinear effects) [41]. In comparison, in two-sample Mendelian randomization, there is a risk of bias if the exposure/outcome relationship varies between the two samples used to obtain the necessary effect estimates. Furthermore, in one-sample Mendelian randomization, compared with two-sample MR, it is possible to avoid bias resulting from having to use summary data (in two-sample MR) that has been conditioned on other variables that can result in collider bias [42]. An important strength of the two-sample Mendelian randomization approach is the number of sensitivity analyses developed to explore potential presence of bias due to horizontal pleiotropy [41]. In the presence of weak instrument bias, estimates from one-sample Mendelian randomization will be biased towards the multivariable regression (observational) estimate and in the presence of residual confounding may be biased (commonly away from the null) [41]. By contrast, the estimate from the two-sample Mendelian randomization will be biased towards the null [41]. The statistical analyses were conducted using Stata version 15 (StataCorp, Texas) and R version 3.5.1 (R Foundation, www.R-project.org). The analysis code is provided in Additional file 2.

## Results

The mean age at menarche in UK Biobank was 12.9 years (standard deviation 1.6 years). The main GRS (GRS_-all_), including 360 SNPs, explained 6.1% of the variation in age at menarche (*F*-statistic = 1043) (Additional file 3: Table S1). Steiger filtering found that 27 of the 360 SNPs included in the GRS for age at menarche explained more of the variation in adult BMI than age at menarche. The genetic risk score including 333 SNPs after Steiger filtering (GRS_-Steiger_) explained 5.8% of the variation in age at menarche (*F*-statistic = 979). After excluding SNPs associated with childhood BMI, the GRS including the remaining 353 SNPs (GRS_-child BMI_) explained 6.0% of the variance in age at menarche (*F*-statistic = 1012), and after excluding childhood and/or adult BMI associated SNPs, the GRS including the remaining 154 SNPs (GRS_-BMI_) explained 3.0% of the variance (*F*-statistic = 489). The main GRS (GRS_-all_) was not associated with study center or genotyping chip after adjusting for the first 10 genetic principal components (*P* value 0.2 for chip and ≥ 0.6 for study center). There was no strong evidence of a relationship between the GRS_-all_ and age at recruitment (*P* value 0.4). The linear correlation coefficient was − 0.002.

**Fig. 3:**
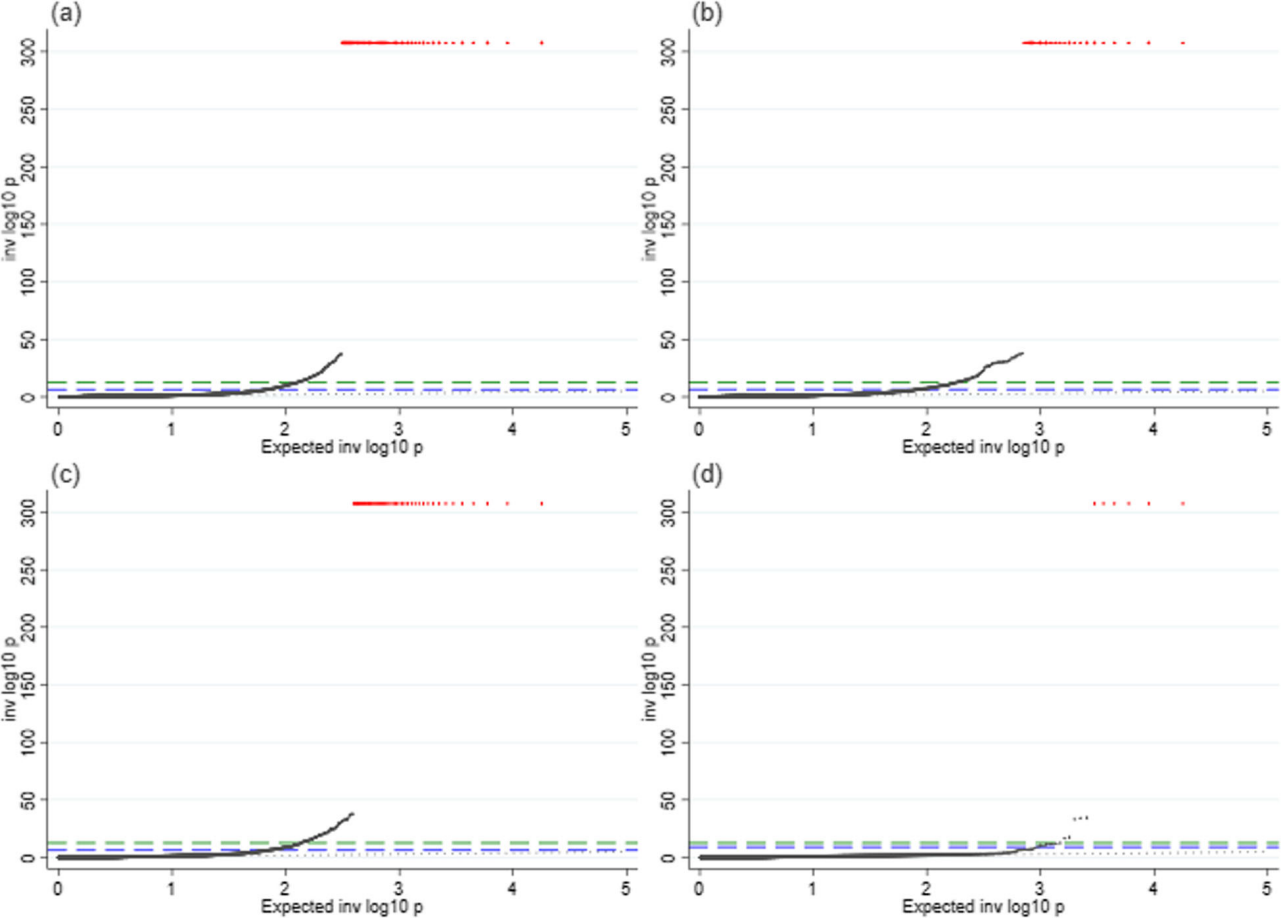
QQ-plots for the Mendelian randomization analysis of age at menarche in relation to 17,893 traits. **a** Main analysis (GRS_-all_). **b** Sensitivity analysis excluding SNPs that explained more of the variation in BMI than age at menarche (GRS_-Steiger_). **c** Sensitivity analysis excluding SNPs associated with childhood BMI (GRS_-child BMI_). **d** Sensitivity analysis excluding SNPs associated with childhood and/or adult BMI (GRS_-any BMI_). Green dashed line: Bonferroni-corrected threshold (*P* value ≤ 2.79 × 10^−6^). Blue dashed line: false discovery rate threshold (*P* value ≤ 1.73 × 10^−3^ for analysis using GRS_-all_; *P* value ≤ 1.61 × 10^−3^ for the analysis using GRS_-Steiger_; *P* value ≤ 1.68 × 10^−3^ for analysis using GRS_-child BMI_; *P* value ≤ 1.03 × 10^−4^ for analysis using GRS_-any BMI_). Black dotted line: actual = expected. Black points: results of tests performed in MR-pheWAS. Red stars: results with *P* values < 2.23 × 10^−308^

**Fig. 4:**
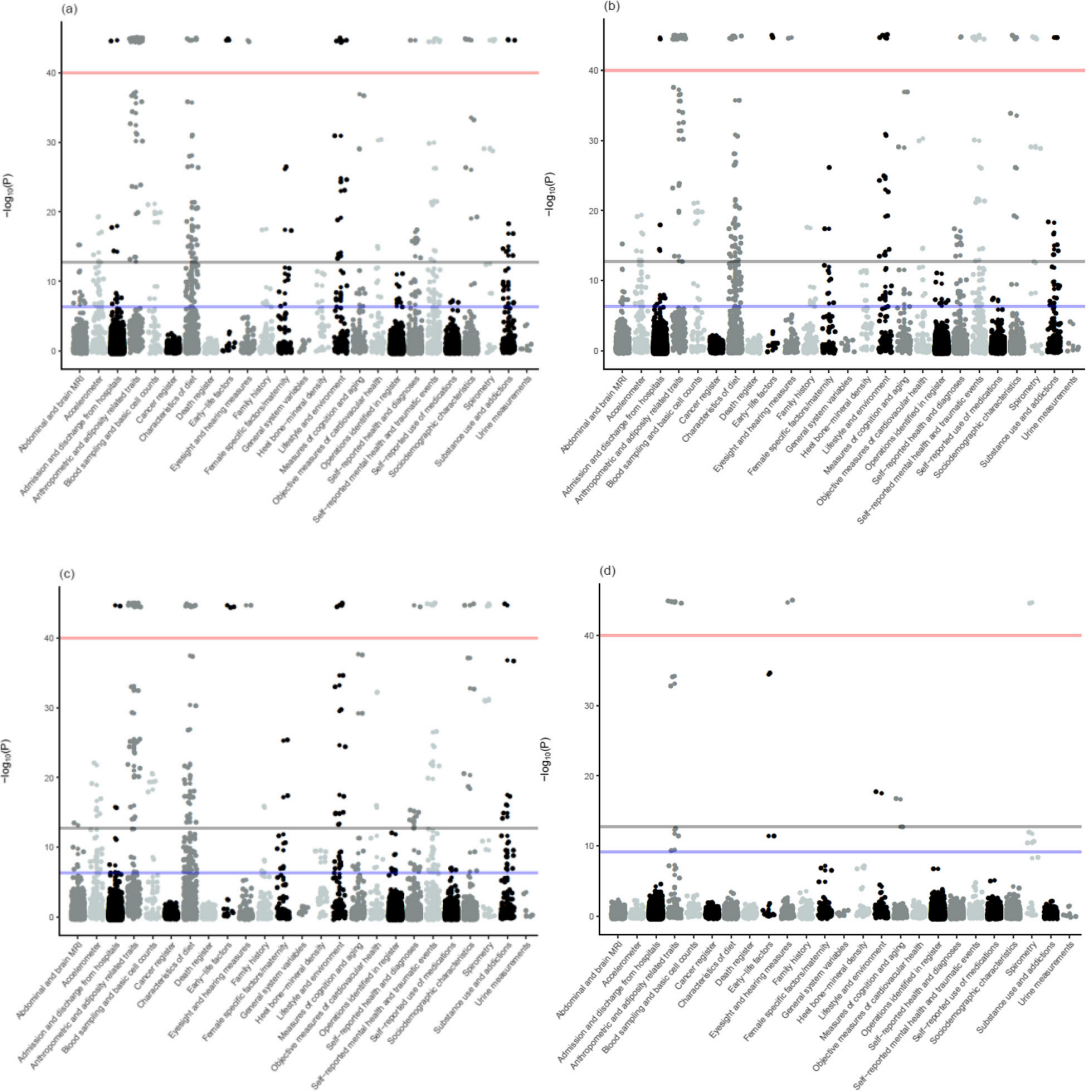
Manhattan plot of results for Mendelian randomization analysis of age at menarche in relation to 17,893 traits. **a** Main analysis (GRS_-all_). **b** Sensitivity analysis excluding SNPs that explained more of the variation in BMI than age at menarche (GRS_-Steiger_). **c** Sensitivity analysis excluding SNPs associated with childhood BMI (GRS_-child BMI_). **d** Sensitivity analysis excluding SNPs associated with childhood and/or adult BMI (GRS_-any BMI_). Gray line: Bonferroni-corrected threshold (*P* value ≤ 2.79 × 10^−6^). Blue line: false discovery rate threshold (*P* value ≤ 1.73 × 10^−3^ for analysis using GRS_-all_; *P* value ≤ 1.61 × 10^−3^ for the analysis using GRS_-Steiger_; *P* value ≤ 1.68 × 10^−3^ for analysis using GRS_-child BMI_; *P* value ≤ 1.03 × 10^−4^ for analysis using GRS_-any BMI_). All findings above the red line indicate results that have *P* values smaller than the limit for what is quantified in R software (*P* value < 2.23 × 10^−308^)

Of the 17,893 tests performed, our MR-pheWAS analysis (using GRS_-all_) identified potential effects of age at menarche on 619 traits (3.5% of all traits) at a false discovery rate of 5% (*P* value ≤ 1.73 × 10^−3^), and 295 (1.6% of all traits) potential effects when using the more stringent Bonferroni-corrected threshold (*P* value ≤ 2.79 × 10^−6^). A quantile-quantile plot of *P* values is shown in Fig. 3a. The distribution of findings across categories of traits is shown in Fig. 4a, while a detailed list describing the findings and the direction of the effects is provided in Additional file 3: Table S2. Of the 619 potential effects of age at menarche, 88 were on BMI and other anthropometric traits, 111 were potential effects on diet, and 29 captured measures of physical activity. We also noted potential effects of younger age at menarche on lower lung function, greater heel BMD, increased risk of psychosocial/mental health issues, poorer cognition, increased report of physical health problems, and measures of reproductive health.

For the reproductive health indicators, we observed potential effects of younger age at menarche on younger age at first birth, younger age at last birth, earlier use of exogenous hormones (both oral contraceptives and hormone replacement therapy), lower risk of bilateral oophorectomy, higher likelihood of pregnancy terminations, lower risk of miscarriage, and higher likelihood of having already experienced menopause. Regarding specific health outcomes, we identified potential effects of younger age at menarche on greater risk of cardiometabolic outcomes (type 2 diabetes, hypertension, ischemic heart disease, cerebral infarction, and angina). Other potential effects of younger age at menarche included increased risk of self-reported osteoporosis, arthrosis, osteoarthritis, and cholecystitis. More novel findings included potential effects of younger age at menarche on increased risk of neuroticism, sleep disorders/chronotype, and having been sexually abused during childhood. Results of our sensitivity MR-pheWAS analysis adjusting for genotyping chip were similar to the main analysis, with 617 findings below a 5% false discovery rate *P* value threshold and 295 below a Bonferroni-corrected *P* value threshold, with similar traits identified in these and the main analysis (Additional file 3: Table S3).

The sensitivity analysis using Steiger filtering to exclude SNPs that explained more of the variation in BMI from the genetic risk score yielded a slightly smaller number of findings, with 576 at the 5% false discovery rate threshold, out of which 245 were below the Bonferroni-corrected *P* value threshold (Figs. 3b, 4b, Additional file 3: Table S4). A total of 207 of the 245 findings that were below the Bonferroni-corrected *P* value threshold after Steiger filtering also passed this threshold in the main analysis.

Overall, the results were also similar when we excluded SNPs associated with childhood BMI from the GRS for age at menarche (GRS_-child BMI_), yielding 601 significant findings below the 5% false discovery rate (*P* value ≤ 1.68 × 10^−3^), out of which 290 were below the Bonferroni-corrected threshold, and the outcomes showing potential effects consistent with those in the main analysis (Figs. 3c, 4c, and Additional file 3: Table S5).

When we excluded all SNPs associated with childhood or adult BMI from the GRS for age at menarche (GRS_-BMI_), we identified 37 potential effects of age at menarche below false discovery rate (*P* value ≤ 1.03 × 10^−4^), out of which 29 were below the Bonferroni-corrected threshold (Figs. 3d, 4d, and Additional file 3: Table S6). These 37 identified relationships were a subset of those identified in our main analyses (GRS_-all_). Despite the exclusion of all SNPs associated (at a genome-wide significant level) with BMI, we still observed a potential effect of younger age at menarche on higher adult BMI (at a mean age of 57), lower height, lower lung function, greater BMD, and higher risk of hypertension. We also found a potential effect of younger age at menarche on younger age at first sexual intercourse, younger age of starting to use oral contraceptives, and younger age at first delivery among others.

**Fig. 5:**
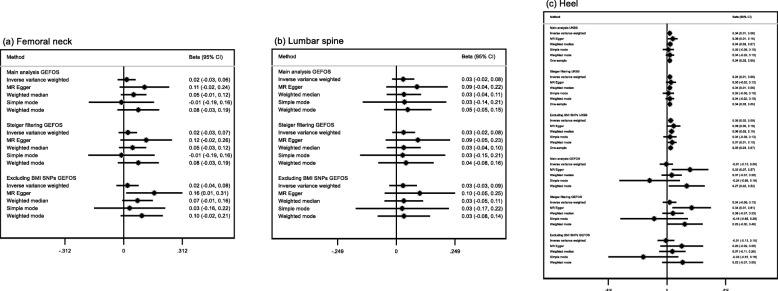
Estimates of the potential causal effect of age at menarche on bone-mineral density. BMD, bone-mineral density; BMI, body mass index; CI, confidence interval; UKBB, UK Biobank. The results reflect standard deviation difference in BMD measurements per year decrease in age at menarche. The BMD measurements were standardized by age, weight, height (heel BMD only), and genomic principal components. The measurement of femoral neck BMD was available for 22,990 women of European ancestry from the GEFOS consortium, lumbar spine BMD was available for 22,177 women of European ancestry from the GEFOS consortium, and heel BMD was available for 4566 individuals of European ancestry. For the GEFOS consortium, the main analysis of femoral and lumbar spine BMD included 263 autosomal SNPs in the genetic risk score for age at menarche, while the main analysis of heel BMD included 252 SNPs. The sensitivity analysis of femoral and lumbar spine BMD excluding BMI-related SNPs included 166 SNPs in the genetic risk score for age at menarche, while the sensitivity analysis of heel BMD included 158 SNPs

We attempted to replicate some of our findings in independent samples. For ease of comparability, we show the effect estimates from an instrumental variable analysis in UK Biobank using the same standardization/adjustment strategy applied in the published GWAS studies (see Additional file 1 for details). We also show the results for UK Biobank only adjusting for age and the first 10 genetic principal components, for BMD and lung function (Additional file 4: Figure S1 and S2, respectively). For heel BMD, we found little evidence of a difference between the estimates in UK Biobank and GEFOS, although the confidence intervals in GEFOS were wide and included the null value (Fig. 5; *I*^2^ heterogeneity statistic *P* values ≥ 0.08). We observed little evidence for an increase in both femoral neck and lumbar spine BMD per year decrease in age at menarche in GEFOS as all confidence intervals included the null (Fig. 5). The intercept from the MR-Egger regression was consistent with the null (no directional horizontal pleiotropy) for all BMD replication analyses except the main analysis (GRS_all_) of heel BMD in GEFOS (Additional file 3: Table S7; MR-Egger intercept *P* value = 0.01).

**Fig. 6:**
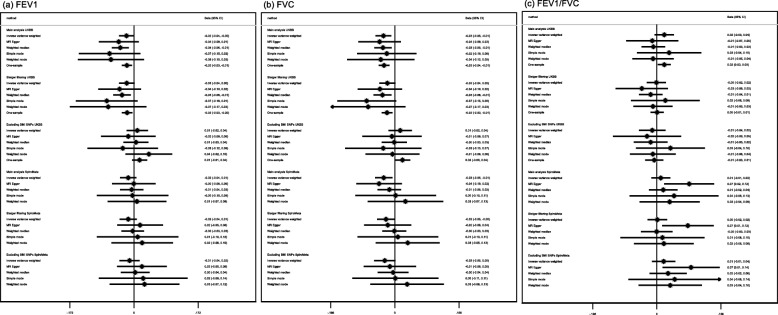
Estimates of the potential causal effect of age at menarche on adult standardized lung function measurements. BMI, body mass index; CI, confidence interval; FEV_1_, forced expiratory volume at 1 s; FVC, forced vital capacity; UKBB, UK Biobank. The results display the change in the ranked-based inverse normal transformed spirometry measurements per year decrease in age at menarche. The spirometry measurements were standardized by age, height, smoking status, and genomic principal components. The analysis in SpiroMeta included 79,055 individuals of European ethnicity. For the SpiroMeta consortium, the main analysis included 328 autosomal SNPs in the genetic risk score for age at menarche, while the sensitivity analysis excluding all SNPs related to childhood and/or adult BMI included 200 autosomal SNPs

**Fig. 7:**
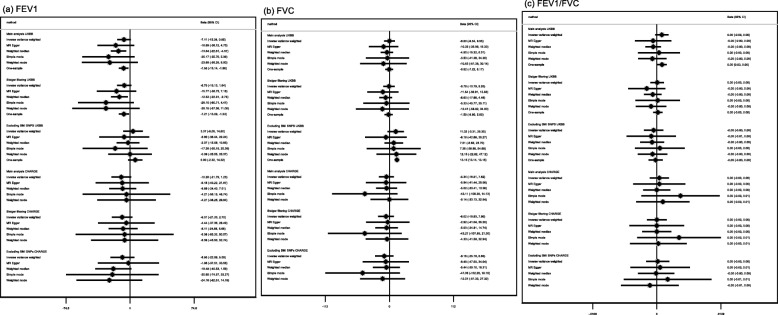
Estimates of the potential causal effect of age at menarche on adult raw lung function measurements. BMI, body mass index; CI, confidence interval; FEV_1_, forced expiratory volume at 1 s; FVC, forced vital capacity; UKBB, UK Biobank. The results display the change in milliliters in the spirometry measurements (FEV_1_ and FVC), or change in the proportion airway obstruction (FEV_1_/FVC), per year decrease in age at menarche. The estimates are adjusted for age, height, smoking status, and genomic principal components. The analysis of the CHARGE consortium included 60,552 individuals of European ethnicity. For the CHARGE consortium, the main analysis included 350 autosomal SNPs in the genetic risk score for age at menarche, while the sensitivity analysis excluding all SNPs related to childhood and/or adult BMI included 213 autosomal SNPs

**Fig. 8:**
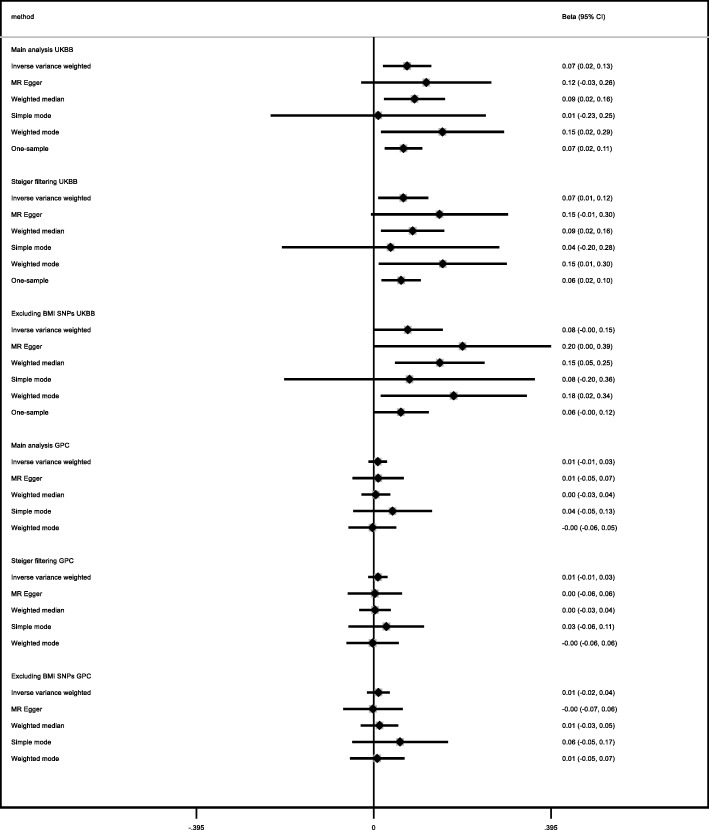
Estimates of the potential causal effect of age at menarche on neuroticism. BMI, body mass index; CI, confidence interval; GPC, Genetics of Personality Consortium; UKBB, UK Biobank. The estimates reflect the change in the harmonized neuroticism score per year decrease in age at menarche adjusted for age and principal components. The Genetics of Personality Consortium (GPC) analysis included 63,661 individuals. For the GPC consortium, the main analysis included 344 SNPs in the genetic risk score for age at menarche, while the sensitivity analysis excluding SNPs associated with BMI included 208 SNPs

**Fig. 9:**
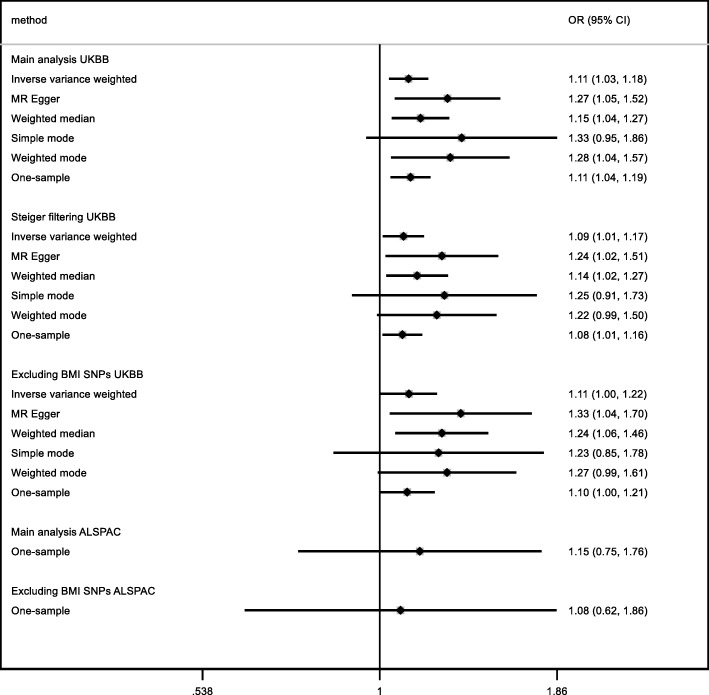
Estimates of the potential causal effect of age at menarche on risk of sexual abuse. ALSPAC, Avon Longitudinal Study of Parents and Children; BMI, body mass index; CI, confidence interval; UKBB, UK Biobank. The ordinal response scale to sexual abuse in UK Biobank was converted to a binary variable denoting whether the participant reported any history of childhood sexual abuse, to be comparable to the replication cohort. The estimates reflect the change in risk of sexual abuse per year decrease in age at menarche after adjusting for 5 principal components. The analysis of the Avon Longitudinal Study of Parents and Children (ALSPAC) cohort included 5953 women. For ALSPAC, the main analysis included 342 SNPs in the genetic risk score for age at menarche, while the sensitivity analysis excluding SNPs associated with BMI included 208 SNPs

For FEV_1_ and FVC, the potential effects of age at menarche in UK Biobank and the replication cohorts (SpiroMeta and CHARGE) were consistent, although the majority of confidence intervals in replication analyses included the null value (Figs. 6 and 7; all *I*^2^ heterogeneity statistic *P* values ≥ 0.07). We observed no strong evidence of an effect of age at menarche on the FEV_1_/FVC ratio (Figs. 6 and 7). The intercepts from the MR-Egger regression for the lung function measures were consistent with the null (Additional file 3: Table S7; *P* values > 0.2), except for FEV_1_/FVC in SPIROMETA (*P* value = 0.04). There was no strong evidence to support an effect of age at menarche on neuroticism in the GPC consortium (Fig. 8), although estimates were largely consistent with those in UK Biobank (all *I*^2^ heterogeneity statistic *P* values ≥ 0.14 except for weighted median approach for which *I*^2^*P* value = 0.02). The MR-Egger intercept for the regression of age at menarche on neuroticism in the GPC consortium also indicated evidence of unbalanced directional pleiotropy (Additional file 3: Table S7; *P* value 0.02). In the Avon Longitudinal Study of Parents and Children, the GRS for age at menarche explained 7.6% of the variation in age at menarche (Additional file 3: Table S8). The results from our replication analysis of childhood sexual abuse were consistent with the results in UK Biobank (*I*^2^ heterogeneity statistic *P* value = 0.9), although the confidence interval was very wide and included the null value (Fig. 9). The MR-PRESSO results from the global test for horizontal pleiotropy and the distortion test for all of the relationships evaluated in the follow-up analyses (presented in Additional file 3: Table S9) found little evidence of horizontal pleiotropy, as none of the effect estimates examined showed both global and distortion tests with *P* values < 0.05. We therefore do not present the causal effect estimates after adjustment for outliers.

The genetic risk scores for adult BMI and height explained 6% and 27% of the variation in these traits in UK Biobank, respectively (Additional file 3: Table S10). Using multivariable Mendelian randomization, we found evidence of an independent effect of earlier menarche on heel BMD, FEV_1_, FVC, and neuroticism after accounting for adult BMI, but little evidence of an independent effect on risk of childhood sexual abuse and obstructive lung disease (Additional file 3: Table S11). There was little evidence to support a relationship between age at menarche and lung function independently of height (Additional file 3: Table S11), with effect estimates attenuating towards the null.

## Discussion

We found that younger age at menarche has a potential effect on a broad range of health-related traits. When we accounted for the genetic overlap between age at menarche and BMI, the number of potential effects identified in our MR-pheWAS at a 5% false discovery rate dropped from 619 to 37, though whether removing all BMI-related SNPs reduced bias is unclear as this could be removing a potential causal path between age at menarche and the outcome that is mediated by adult BMI. In relation to this, the majority of the potential causal effects (576/619) remained when we removed the genetic variants that explained more of the variation in BMI than age at menarche (the variants most likely to reflect horizontal pleiotropic pathways).

We conducted replication and follow-up analyses to test Mendelian randomization assumptions following our MR-pheWAS for four of the potential effects identified. While the estimated effects of younger age at menarche on lower lung function and higher BMD were consistent in UK Biobank and the replication cohorts, our replication analyses were undertaken in smaller samples than UK Biobank and had limited statistical power particularly for the binary childhood sexual abuse. This meant that with the exception of an effect of younger age at menarche with higher BMD, the confidence intervals for the replication estimates included the null. Additional replication studies of these findings are therefore necessary when larger study populations (or published GWAS studies) of these outcomes become available. Future studies should also follow-up other potential effects identified in our MR-pheWAS which we did not follow-up to estimate the magnitude of the causal effects and investigate validity of Mendelian randomization assumptions.

In common with most existing Mendelian randomization studies, including previous studies of the effects of age at menarche on hypothesized outcomes [9, 11, 12, 14–20], we did not explore potential nonlinear effects of age at menarche on outcomes. The reason we have not done this and it is rarely done in other studies is because current methods are only feasible in one-sample MR and require very large sample sizes, and the choice of where to put thresholds (for examining MR effects in subsets across the distribution) is unclear [43]. As methods are further developed, including for potential use in two-sample MR, this could be further explored in future studies that follow up specific findings from our MR-PheWAS.

Due to the number of potential effects on health-related traits by age at menarche identified in the MR-PheWAS, we could not follow-up all the findings with sensitivity and replication analyses. Several other health-related traits were related to the genetic risk score for age at menarche in our MR-pheWAS, and their causal relationship with age at menarche should be further explored in future studies. An underlying genetic predisposition to younger age at menarche was for example linked to a younger age at first birth, higher number of offspring, higher risk of miscarriages/stillbirths, and higher risk of sleeplessness/insomnia, depression, osteoarthritis, arthrosis, and cholecystitis among others. Our MR-PheWAS included some early-life health-related outcomes that predate menarche (maternal smoking around the time of delivery, comparative body size at age 10, and whether the woman was breastfed). We note that the MR-PheWAS estimates the association of a genetic predisposition to younger age at menarche with these outcomes, and this does not necessarily reflect an effect of age at menarche on these outcomes since there may have been violations in the instrumental variable assumptions. We chose to follow-up childhood sexual abuse, but we were not able to distinguish whether the abuse occurred before or after menarche, as detailed information on the particular ages during which the abuse occurred was not available. Considered in the context of a genetic predisposition to earlier maturation (age at menarche occurs at a specific time but pubertal changes will have started earlier), the potential effect we see here may also reflect a tendency for girls who undergo puberty earlier to be more prone to abuse.

One limitation of our analysis is the low response in UK Biobank (5%), which could have resulted in selection bias [44, 45]. Participants in UK Biobank have been shown to be healthier and of a higher socioeconomic position compared to other estimates for the British population [46]. This could have resulted in a lower burden of some of the health-related outcomes evaluated, such as a lower proportion of smokers, lower mean BMI, lower overall CVD risk (less diabetes and less hypertension), and less psychological problems, among others. The effect of potential selection bias likely varies across the large number of health-related outcomes evaluated. Reassuringly, the mean age at menarche was as expected based on the estimated age at menarche in the general population [47]. Recruitment into the cohort is also restricted to individuals who had survived until the age at which time they were recruited, meaning that if age at menarche is causally related to (premature) mortality, survival bias may have influenced our findings, as it would have in any previous prospective studies.

A second limitation is the statistical power of our replication analyses. We conducted post hoc power calculations to evaluate the power of our replication analyses. Assuming a type 1 error rate of 5%, and that the instrument explains 5% of the variation in age at menarche, we calculated the minimal effect detectable with 80% power (Supplement Table S12). The effect estimates reflect the change in the standard deviation of the continuous outcomes per standard deviation decrease in age at menarche. These results indicated that we were adequately powered (80%) to detect a standard deviation in the continuous outcomes ranging from 0.04 for the lung function measures in the CHARGE consortium to 0.17 for heel bone-mineral density in the GEFOS consortium. We were further powered to detect a twofold increase in the odds of sexual abuse per standard deviation increase in age at menarche in Avon Longitudinal Study of Parents and Children. The replication analyses were therefore powered to detect modest effect estimates similar to that observed in UK Biobank, with the exception of the analysis in Avon Longitudinal Study of Parents and Children. Thus, for this outcome in particular, further replication in larger studies is necessary. To our knowledge, we have used the largest sample sizes currently available for replication (i.e., the largest samples with the relevant outcome and genetic data).

A strength of our study is the use of genetic variants as instrumental variables to reduce the risk of confounding. However, the weights we used to generate our GRS included a subpopulation of women participating in UK Biobank. This overfitting of the estimated coefficients for the relationship between the SNPs and age at menarche to UK Biobank could therefore have contributed to an overestimation of the amount of variation in age at menarche explained by the GRS. On the other hand, the known “winner’s curse” in GWAS studies might also lead to an underestimation of causal effect estimates using Mendelian randomization [48]. We attempted to minimize the risk of bias due to population stratification by restricting to individuals of European ethnicity and adjusting for genetic principal components. However, this limits the generalizability of our results to people from other backgrounds. A core Mendelian randomization assumption states that the genetic instruments should only affect the outcome through pathways that are via the exposure of interest (which may be violated by horizontal pleiotropy). The GRS for age at menarche includes a large number of genetic variants. This increases the likelihood that horizontal pleiotropy may be biasing our results. However, we estimated causal effects on our follow-up analyses using two-sample Mendelian randomization methods that require different assumptions about the extent that the exclusion restriction can be violated (e.g., the extent that horizontal pleiotropy can occur) and tested for unbalanced horizontal pleiotropy using MR-Egger regression and found little evidence of this for these outcomes. The self-reported information used to create a score for neuroticism and to define a history of childhood sexual abuse did not directly compare across UK Biobank and the replication cohorts. However, the point estimates particularly for sexual abuse were remarkably similar.

Due to the genetic correlation between age at menarche and BMI, we repeated the analyses excluding SNPs associated with BMI at a Bonferroni-corrected level from the age at menarche GRS. Notably, the GRS_-BMI_ for age at menarche excluding SNPs associated with childhood and adult BMI was still associated with adult BMI (the PHESANT estimated change in the inverse ranked transformed BMI per allele increase was 0.006 (95% CI, 0.005, 0.006) for GRS_-all_, while it was 0.002 (95% CI, 0.002, 0.003) for GRS_-BMI_). This is likely due to the presence of SNPs with modest effects on BMI that did not reach the Bonferroni-corrected *P* value threshold in the published GWAS of childhood and adult BMI. Steiger filtering indicated that only 27 of the 360 SNPs included in the GRS for age at menarche explained more of the variation in BMI than age at menarche. The low proportion of the GRS SNPs that explained a greater proportion of variation in BMI than age at menarche suggests that most of the SNPs are valid instruments for age at menarche and their relationship with BMI is downstream of age at menarche (reflecting that BMI is a potential mediator of the effects of interest). This is why we present the findings from the genetic risk score including all genetic variants as the main analysis. We conducted multivariable Mendelian randomization in UK Biobank, which also suggested effects of age at menarche on heel BMD, FEV1, FVC, neuroticism, and childhood sexual abuse independent of BMI. In our replication analysis of lung function and neuroticism, we had to use estimates of the effect of the SNPs on the outcomes including both sexes, as GWAS results of these traits by sex were not available. However, there is evidence that a large proportion of the SNPs included in the GRS for age at menarche also predict puberty development in a similar way in males (i.e., those predicting early pubertal development in females also reflect an earlier puberty development in males) [16]. We did not conduct multivariable Mendelian randomization for the entire MR-PheWAS, as MR-pheWAS is hypothesis-generating, identifying potential effects which subsequently need to be followed up with further analyses. Multivariable mendelian randomization also has some limitations, as it requires the variables used in the model to have similar instrument strength [49]. The stronger instrument strength for BMI compared with age at menarche could therefore have attenuated our findings and caused us to miss potential causal effects of age at menarche. PHESANT performs the test of the association of a genetic instrumental variable with a wide range of outcomes (here ~ 18,000 outcomes) to identify outcomes for which there is evidence of a potential causal effect, providing the opportunity to identify novel causal effects. It was not possible for us to complete replication and assumption testing for all of the potential effects identified (over 600 at a 5% false discovery rate), and we therefore followed up 4 selected examples identified in our MR-pheWAS to estimate the magnitude of the causal effect and explore validity of instrumental variable assumptions. Thus, a useful further advance may be automation of follow-up analyses and replications, for example, using a system like MR-Base. The lack of available data to undertake replication and assumption testing for all outcomes will remain an issue, but with increasing amounts of GWAS studies, this will improve over time.

We found some evidence of an effect of younger age at menarche on lower lung function in UK Biobank, but while the confidence intervals were consistent in both the SpiroMeta and CHARGE consortia replication samples, the majority of confidence intervals included the null. Previously, a Mendelian randomization analysis of age at menarche and lung function using data from the earlier release of the genetic data from UK Biobank, where UK Biobank was 1 of 3 cohorts used to estimate the effect on adult lung function (total *n* = 46,944 women), also reported lower adult FEV_1_ and FVC with younger age at menarche [18]. One potential explanation for this finding is that younger age at menarche/earlier exposure to female sex hormones terminates lung growth at a younger age, and subsequently leads to a lower maximally attained lung function and lower lung function in adulthood [50]. This potential explanatory mechanism was further supported by the fact that we observed little evidence to support a relationship between age at menarche and lung function after accounting for adult height in multivariable Mendelian randomization.

Our results in UK Biobank suggest that younger age at menarche may result in greater adult heel BMD, but while our replication estimates were largely consistent with our UK Biobank results, confidence intervals were wide and included the null. A beneficial effect of age at menarche on BMD, if one exists, would be consistent with results of a recent Mendelian randomization study indicating that a delayed onset of puberty may causally affect fracture risk [20]. The importance of female sex hormones for bone health is established and is clearly reflected in the reduction of bone mass and increased risk of osteoporosis among postmenopausal women [51].

Our results in UK Biobank also suggested that younger age at menarche might result in higher levels of neuroticism. Notably, a previously published BMI MR-pheWAS supported a causal effect of BMI on neuroticism [22]. Any potential explanatory mechanisms for a relationship between age at menarche and neuroticism are less evident than for lung function and BMD and need to be further investigated. We also observed evidence of a higher risk of sexual abuse among women with a younger age at menarche, although the replication analysis was underpowered. This association has been previously reported [52–54].

The main contribution of our study is to increase understanding of potential effects of age at menarche on health-related traits. While it is not possible to directly intervene on age at menarche, it is important to understand the causal pathways to disease and the role that pubertal timing might play. Our study therefore lays the foundations for future research into the biosocial mechanisms that might explain how age at menarche might influence the health-related traits, some of which are likely to be modifiable. As several of the health-related traits are common and complex with multiple risk factors, if our findings are further replicated in larger cohorts providing more precise estimates, they can be used to give advice on risk. For example, if it is established that younger age at menarche is causally related to lower lung function, girls with younger age at menarche might be advised to remain physically fit to maintain their lung function. Furthermore, knowing that you have one (unmodifiable) risk factor for some health outcomes is useful for women, as it means they should attempt to avoid any additional adverse modifiable risk factors.

## Conclusions

Our results suggest that younger age at menarche has potential effects on a broad range of health-related traits. Follow-up analysis indicated imprecise evidence of an effect of younger age at menarche on higher bone-mineral density, lower lung function, greater score for neuroticism, and greater risk of childhood sexual abuse in the smaller replications samples available, and these relationships should therefore be re-examined when larger study populations become available. Future studies are needed to further investigate the potential effects which we did not follow-up here. Additional studies using other designs with different biases and sufficient statistical power to replicate our findings would also be useful. Where future studies provide strong evidence for causal effects of age at menarche on several outcomes, studies to explore potential modifiable mechanisms to mitigate any effect of age at menarche will be important.

## Supplementary information

**Additional file 1.** Online Methods.

**Additional file 2.** Analysis code.

**Additional file 3:Table S1.** Strength of the genetic instrument in predicting age at menarche in UK Biobank. **Table S2.** Findings from the main analysis of the genetic risk score for age at menarche. **Table S3.** Finding from the main analysis of the genetic risk score for age at menarche after additional adjustment for chip used for the genotyping. **Table S4.** Finding from the main analysis of the genetic risk score for age at menarche after Steiger filtering. **Table S5.** Findings from the sensitivity analysis of the genetic risk score for age at menarche excluding SNPs associated with childhood body-mass index. **Table S6.** Findings from the sensitivity analysis of the genetic risk score for age at menarche excluding SNPs associated with childhood and/or adult body-mass index. **Table S7.** MR-Egger intercept. **Table S8.** Results from MR-PRESSO global test for pleoitropy and distortion tests. **Table S9.** Strength of the genetic instrument in predicting age at menarche in ALSPAC. **Table S10.** Strength of the genetic instrument in predicting body-mass index and height in UK Biobank. **Table S11.** Findings from the multivariable mendelian randomization analysis further adjusting for body-mass index and height. **Table S12.** Post hoc power calculation analyses for the replication analyses.

**Additional file 4:Figure S1.** Estimates of the potential causal effect of age at menarche on bone-mineral density in UK Biobank. **Figure S2.** Estimates of the potential causal effect of age at menarche on adult lung function in UK Biobank.

## Data Availability

The data underlying the results presented in this study can be made available on request from the UK Biobank. Questions and applications for access to data can be submitted to access@ukbiobank.ac.uk.
