# Supplementary material for: Identifying potential causal effects of age at menarche: a Mendelian randomization phenome-wide association study
Source: BMC Med. 2020 Mar 23;18:71. doi: 10.1186/s12916-020-01515-y (PMC7087394; doi:10.1186/s12916-020-01515-y)
Supplement: Supplementary file 1 — Additional file 1. Online Methods. [file 12916_2020_1515_MOESM1_ESM.docx]

**Online Methods**

**GEFOS Consortium**

The GEFOS Consortium is a coalition of teams of investigators dedicated to identifying the genetic determi­nants of osteoporosis which includes epidemiological studies across North America, Europe, East Asia and Australia (<http://www.gefos.org/>). In the current study, we only included individuals of European ancestry. All studies were approved by their institutional ethics review committees, and all participants provided written informed consent. Lumbar spine, femoral neck and heel bone mineral density were measured in all cohorts using dual-energy X-ray absorptiometry, following standard protocols. GWAS genotyping was performed by each study following standard protocols, and imputation was then carried out on ~2.5 million SNPs from HapMap [55] Phase 2 release 22 using Genome Build 36. Quality control was performed inde­pendently for each study. To facilitate meta-analysis, each group performed genotype imputation with BIM-BAM,[56] IMPUTE,[57] or MACH,[58] software using genotypes from HapMap Phase 2 release 22, and IMPUTE software was used for imputation. Overall, imputation quality scores for each SNP were obtained from IMPUTE (proper_info) and MACH (rsq_hat) statistics.

Each study performed genome-wide associa­tion analysis for femoral neck bone-mineral density and lumbar spine bone-mineral density, using sex-specific and age-, weight- and genomic principal component–adjusted standardized residuals analyzed under an additive (per allele) genetic model. The heel bone-mineral density measures were standardized by sex and adjusted for age, age squared, height, weight, machine type and genomic principal components. The analysis of imputed genotype data accounted for uncertainty in each genotype prediction by using either the dosage information from MACH or the genotype probabilities from IMPUTE and BIM-BAM. Studies used MACH2QTL,[58] directly or via GRIMP,[59] (which uses genotype dosage value as a predictor in a linear regression framework), SNPTEST,[57] Merlin,[60] BIM-BAM or the linear mixed-effects model of the Kinship and ProbABEL [61]. The imputed genotypes were coded as continuous variables from 0 to 2 to take into account imputation uncertainty. The genomic control method was used to correct the standard error (SE) by the square root of the genomic inflation factor (λ): SEcorrected = s.e.m. × √λ [62]. Inverse-variance fixed-effects meta-analysis (using METAL software) was conducted with double genomic correction,[63] to control for potential inflation of the test statistics in individual studies and in the meta-analysis.

For the current analysis, a total of 22,990 women of European ancestry and 263 genetic variants were available for the analysis of femoral neck bone-mineral density, 22,177 women of European ancestry and 263 genetic variants were available for the analysis of lumbar spine bone-mineral density, while 4,566 individuals of European ancestry and 252 variants were available for the analysis of heel bone-mineral density. Sex stratified results were not available for heel bone-mineral density.

**SpiroMeta**

The SpiroMeta consortium meta-analysis was comprised of a total of 79,055 individuals from 22 studies [35]. Thirteen studies (n=21,436 individuals) were imputed to the 1000 Genomes Project Phase 1 reference panel (B58C [T1DGC and WTCCC],[64] BHS1&2, three Croatian studies [CROATIA-Korcula, CROATIA-Split and CROATIA-Vis], Health 2000, KORA F4, KORA S3, LBC1936, NSPHS, ORCADES, SAPALDIA and YFS and 9 studies (n=61,682 individuals) were imputed to the Haplotype Reference Consortium (HRC) panel (EPIC [obese cases and population-based studies],[65] GS:SFHS, NFBC1966, NFBC1986, PIVUS, SHIP, SHIP-TREND, UKHLS and VIKING). The individual studies all obtained written informed consent and ethical approval from local ethical committees. The current study only included individuals of European ancestry.

In each study, linear regression models were fitted for each lung function trait (FEV1, FEV1/FVC and FVC), with adjustment for age, age squared, sex and height. For studies with unrelated individuals, these models were fitted separately in ever smokers and never smokers, with additional adjustment for principal components of ancestry. Studies with related individuals fitted mixed models in all individuals to account for relatedness, with ever smoking status as a covariate. In all studies, rank-based inverse normal transformations were undertaken on the residuals, with these transformed residuals used as the phenotype for association testing under an additive genetic model. In the study level results, variants were excluded if they had a very low minor allele count or imputation quality (info) <0.3. In studies with unrelated individuals, the ever and never smokers results were combined, using inverse variance weighted meta-analysis, to give an overall study result. Genomic control was then applied to all study level results, before combining results across all studies using inverse variance weighted meta-analysis. LD score regression intercepts for the meta-analysis were close to 1 and so genomic control was not applied.

For the current analysis, 79,055 individuals of European ancestry and 328 genetic variants were included. Sex-stratified results were not available.

**CHARGE**

Details of the CHARGE Consortium meta-analysis of 1000 Genomes imputed variants and pulmonary function have been previously published [34]. Briefly, the full meta-analysis included 90,715 individuals from 22 cohorts comprising multiple ancestral populations, including 60,552 individuals of European ancestry from 18 cohorts.

Within each study, linear regression was used to model the additive effect of variants where FEV_1_ and FVC were modeled as milliliters and FEV1/FVC as a proportion, adjusting for age, age^2^, sex, height, height^2^, smoking status, pack-years of smoking, center (if multicenter study), weight (for FVC), and ancestral principal components, including a random familial effect to account for family relatedness when appropriate [66]. Fixed-effects meta-analyses using inverse variance weighting of study-specific results with genomic control correction were then conducted in Meta-Analysis Helper (METAL, <http://www.sph.umich.edu/csg/abecasis/metal/>). Variants with imputation quality scores (r^2^) less than 0.3 and/or a minor allele count less than 20 were excluded from each study prior to meta-analysis. Following meta-analysis, we also excluded variants with less than one-third the total sample size or less than the sample size of the largest study for a given meta-analysis to achieve a minimal sample sizes of 20,184 for European ancestry.

The 350 genetic variants used to create the genetic risk score for age at menarche were looked-up in the CHARGE European meta-analysis results for FEV_1_, FVC, and FEV_1_/FVC.

**GPC**

The Genetics of Personality Consortium (GPC) is a large collaboration of genome-wide associations studies for personality traits [36]. The GWAS study of neuroticism that we used in our two-sample Mendelian randomization analysis included Europeans from 29 different cohorts, where 21 cohorts were from Europe, 6 form the United States, and 2 from Australia. The total combined sample size across the cohorts was 63,661. All cohorts obtained ethical approvals from local institutional review boards and information consent was obtained from all participants. The questionnaire items used to capture neuroticism was harmonized across cohort, and the harmonization process has been described in detail [67]. The different scales used to capture neuroticism across the cohorts included the NEO Personality Inventory, Eysenck Personality Questionnaire, and International Personality Item Pool inventory.

Quality control of the genotype data was performed separately for each cohort using comparable criteria. Specific steps included evaluation of European ancestry, sex-inconsistencies, mendelian errors, and high genome-wide homozygosity. The authors also examined relatedness in samples that aimed to include unrelated individuals. Other checks included Hardy-Weinberg equilibrium, minor allele frequencies, SNP, and sample call rates. The genotype data was imputed using the 1000 Genomes phase 1 version 3 (build 37, hg19) reference panel with software packages such as IMPUTE, MACH, or Minimac. The association analysis in each cohort were conducted using linear regression, adjusting for sex, age and principal components. An additive genetic model was assumed, so that the associations reflected the change in the outcomes per increased copy number of the risk allele. The meta-analysis of the results across the cohorts used a weighted inverse variance approach to combine the cohort-specific results using the METAN software. Sex-specific results were not available.

**ALSPAC**

The Avon Longitudinal Study of Parents and Children (ALSPAC) recruited pregnant women with an expected delivery data between April 1991 and December 1992 living in a defined area of Avon, South West England [37]. The participating rate of invited pregnant women was 75%, resulting in 14,541 participants. Written informed consent was obtained from all participants. Ethical approval for the study was granted by the ALSPAC Law and Ethics Committee and the Local Research Ethics Committees. Please note that the study website contains details of all the data that is available through a fully searchable data dictionary and variable search tool (http://www.bristol.ac.uk/alspac/researchers/our-data/). Self-reported history of childhood sexual abuse was obtained from the women at the time or recruitment. The specific questions asked whether anyone had masturbated in front of them without their consent, whether anyone had sexual intercourse with them without their consent, whether anyone had rubbed their genitals against them without their consent, and whether anyone had tried to put their penis into her mouth without their consent before age 16. The responses to these questions were combined to indicate any sexual abuse.

DNA samples were extracted from whole blood samples taken during pregnancy. The Centre National de Génotypage (Evry, France) carried out DNA genotyping on the Illumina human660W-quad array and genotypes were called with Illumina GenomeStudio. PLINK version 1.07 (<http://pngu.mgh.harvard.edu/purcell/plink>) [68] was used to carry out Quality Control (QC) measures on an initial set of 10,015 subjects and 557,124 directly genotyped single nucleotide polymorphisms (SNPs). SNPs were removed if they displayed more than 5% missing, a Hardy-Weinberg equilibrium (HWE) p-value of less than 1.0x10^-6^, or a minor allele frequency (MAF) of less than 1%. Samples/individuals were excluded if they displayed more than 5% missing values, had indeterminate X chromosome heterozygosity, extreme autosomal heterozygosity, or showed evidence of non-European ancestry. Multidimensional scaling of genome-wide identity was conducted by state pairwise distances using the four HapMap populations as a reference. Autosomal SNPs were imputed using Impute2 v2.2.2 and the 1000 genomes phase 1 version 3 reference panel including 2186 haplotypes from all populations.

For the current analysis, 5,953 women of European ancestry and 342 genetic variants were included. In our one-sample Mendelian randomization analysis, we adjusted for genomic principal components to account for residual population stratification among individuals of European ancestry.

**Outcomes in UK Biobank for the replication analysis**

We chose to further investigate the results for heel bone-mineral density (BMD), lung function, neuroticism and sexual abuse. Heel BMD was measured using the Sahara Clinical Bone Sensometer, which estimates BMD based on an ultrasound measurement of the calcaneus. It does not actually measures BMD, but speed of sound (SOS, in metres/second) and broadband ultrasound attenuation (BUA, in decibels/megahertz). These results are combined to give the Quantitative Ultrasound Index (QUI), or stiffness. From this an estimate is made of BMD (in greams/cm2). We used the raw variable for BMD in our analysis (UK Biobank variable 3148). In order to be able to compare the results from UK Biobank to the findings observed in the two-sample Mendelian randomization analysis of GEFOS, we obtained standardized residuals of the BMD in UK Biobank adjusting for age, age squared, height, weight, and genomic principal components. These standardized residuals were therefore used as the outcome in the instrumental variable analyse (both the one-sample and two-sample analyses).

Forced expiratory volume (FEV1) and forced vital capacity (FVC) were measured at baseline using Vitalograph Pneumotrac 6800 (Vitalograph, UK). Spirometry was not conducted if participants had experienced a chest infection in the last month; had a life-time history of detached retina or collapsed lung; if they had been through a heart attack, eye surgery or surgery to chest or abdomen in last 3months; or if they were currently pregnant or on tuberculosis medications. If the reproducibility of the first two measurements was adequate, defined as a_5% difference in FVC and FEV1, a third measurement was not required. Post-bronchodilator spirometry was not available, although drug treatment was not withheld. We used the highest measure from the array of values of FEV_1_ and FVC (UK Biobank variables 20150 and 20151). We conducted two separate analyses of the lung function measurements in UK Biobank. The first analyses used inverse normal rank transformed residuals of the outcomes, adjusting for age, age squared, sex, height, smoking, and genomic principal components, to be comparable with the SpiroMeta analysis strategy. The second analyses evaluated the lung function measurements on their raw scale, adjusting for age, age^2^, sex, height, height^2^, smoking status, pack-years of smoking, weight (for FVC), and genomic principal components, to be comparable to the adjustment strategy in the CHARGE consortium.

We also evaluated the neuroticism score available in UK Biobank (UK Biobank variable 20127). This score is composed of responses to 12 different questions: Does your mood often go up and down?; Do you ever feel “just miserable” for no reason?; Are you an irritable person?; Are your feelings easily hurt?; Do you often feel “fed-up”?; Would you call yourself a nervous person?; Are you a worrier?; Would you call yourself tense or “highly strung”?; Do you worry too long after an embarrassing experience?; Do you suffer form “nerves”?; Do you often feel lonely?; Are you often troubled by feelings of guilt?. Participants could answer yes, no, don’t know or prefer not to answer. The score summarises the number of yes answers across these twelve score into a single integer score for each participant. The instrumental-variable analysis (one and two-sample Mendelian randomization analysis) in UK Biobank adjusted only for age and genomic principal components.

Finally, we evaluated experience of childhood sexual abuse (UK Biobank variable 20490). Participants were asked “When I was growing up, someone molested me (sexually) ”. The answer options were prefer not to answer, never true, rarely true, sometimes true, often or very often true. Those who responded rarely true, sometimes true, often or very often true were defined as having been sexually abused as a children. The instrumental-variable analyses (one and two-sample Mendelian randomization) adjusted for age and genomic principal components. The GPC consortium did not make any further adjustments in their analyses.

**Multivariable Mendelian randomization in UK Biobank**

We also conducted multivariable Mendelian randomization to examine the role of BMI in the observed relationship between age at menarche and heel BMD, lung function, neuroticism and childhood sexual abuse in UK Biobank [49]. We therefore also generated a weighted genetic risk score for adult BMI for the 940 BMI-related SNPs identified in a recent GWAS available in the imputed data in UK Biobank [30]. To run the multivariable Mendelian randomization analysis, we first estimated the genetically predicted values for age at menarche using linear regression, including the genetic risk scores for age at menarche and BMI as predictors. We subsequently estimated the genetically predicted values for BMI using linear regression, including the genetic risk scores for age at menarche and BMI as predictors. We then examined the relationships of the genetically predicted value for age at menarche with heel BMI, lung function measurements and neuroticism using linear regression, while also adjusting for the genetically predicted values for BMI. The relationship between the genetically predicted value for age at menarche and childhood sexual abuse, after accounting for the genetically predicted value for BMI, was estimated using logistic regression. We corrected the standard errors of the second step using bootstrapping.
