# Supplementary material for: Identifying potential causal effects of age at menarche: a Mendelian randomization phenome-wide association study
Source: BMC Med. 2020 Mar 23;18:71. doi: 10.1186/s12916-020-01515-y (PMC7087394; doi:10.1186/s12916-020-01515-y)
Supplement: Supplementary file 4 — Additional file 4:Figure S1. Estimates of the potential causal effect of age at menarche on bone-mineral density in UK Biobank. Figure S2. Estimates of the potential causal effect of age at menarche on adult lung function in UK Biobank. [file 12916_2020_1515_MOESM4_ESM.docx]

Figure S1 Estimates of the potential causal effect of age at menarche on bone-mineral density in UK Biobank

The results display the change in BMD in g/cm2 per year decrease in age at menarche. The estimates are adjusted for age and genomic principal components.

Figure S2 Estimates of the potential causal effect of age at menarche on adult lung function in UK Biobank

The results display the change in ml in the spirometry measurements (FEV_1_ and FVC), or change in the proportion airway obstruction (FEV_1_/FVC), per year decrease in age at menarche. The estimates are adjusted for age and genomic principal components.
